# 96. Impact of Hospital-Based Pharmacist Discharge Prescription Review on the Appropriateness of Antibiotic Therapy

**DOI:** 10.1093/ofid/ofab466.298

**Published:** 2021-12-04

**Authors:** Amy Spigelmyer, Catessa Howard, Ilya Rybakov, Sheena Burwell, Douglas Slain

**Affiliations:** 1 West Virginia University Medicine, Morgantown, West Virginia; 2 WVU Medicine, Morgantown, West Virginia; 3 West Virginia University School of Pharmacy, Morgantown, West Virginia

## Abstract

**Background:**

Inappropriate antibiotic prescribing upon hospital discharge poses an increased risk of excess costs, adverse drug reactions, readmission, and resistance. Despite high rates of antibiotic prescription errors upon discharge, there is no widely accepted antimicrobial stewardship initiative to prevent such errors. This study evaluated the impact of hospital-based clinical pharmacist discharge prescription review on the appropriateness of antibiotic prescriptions.

**Methods:**

This was a retrospective assessment of patients with discharge antibiotic prescriptions for treatment of pneumonia, urinary tract infections, *Clostridioides difficile* infections, acute skin and skin structure infections (ABSSSI), or Gram-negative bacteremia between January 2019 and July 2020. The two cohorts that were studied were patients on Hospitalist services versus patients on Medicine services, in which only the Medicine services had rounding pharmacists who perform discharge prescription reviews. Outcomes included demographics, appropriateness of therapy, 30-day readmission rates, and error types in discharge prescriptions. Appropriateness of therapy was validated by evidence-based guidelines and three Infectious Diseases-trained pharmacists.

**Results:**

Our study included 300 patients, 150 per cohort. Baseline characteristics were similar between groups, with the exception of increased age (p=0.025) and fewer cases of ABSSSI (p=0.001) in the Hospitalist cohort. A statistically significant higher rate of inappropriateness was seen in the Hospitalist group versus Medicine (pharmacist) group, [69/150 (46% versus 25/150 (17%, respectively (p< 0.00001)]. The difference in appropriateness was mainly driven by pneumonia and UTI prescriptions. Thirty day readmission rates were 17% (26/150) for the Hospitalist cohort versus 11% (16/150) in the Medicine (pharmacist) cohort (p=0.134). The most common prescription error was the duration of therapy.

**Conclusion:**

Appropriateness of antibiotic discharge prescriptions significantly improved in the setting of pharmacist discharge prescription review. This initiative highlights the important role of clinical pharmacists in the setting of outpatient antimicrobial stewardship.

**Disclosures:**

**All Authors**: No reported disclosures

